# ORAL AND ENTERAL NUTRITION THERAPY IN INFLAMMATORY BOWEL DISEASES AMONG THE PEDIATRIC POPULATION: A LITERATURE REVIEW

**DOI:** 10.1590/1984-0462/2020/38/2019032

**Published:** 2020-06-05

**Authors:** Gabriela Neves de Souza, Patrícia Ferrante Draghi, Glauce Hiromi Yonamine

**Affiliations:** a Instituto da Criança do Hospital das Clínicas, Faculdade de Medicina, Universidade de São Paulo, São Paulo, SP, Brazil.; b Centro Universitário Saúde ABC, Faculdade de Medicina do ABC, Santo André, SP, Brazil.

**Keywords:** Inflammatory bowel diseases, Nutritional therapy, Pediatrics, Doenças inflamatórias intestinais, Terapia nutricional, Pediatria

## Abstract

**Objectives:**

To review the literature on oral and enteral nutrition therapy and investigate the evidence of its efficacy as a treatment, as well as in preventing relapses and reducing symptoms of inflammatory bowel diseases in the pediatric population.

**Data source:**

We performed a bibliographic search in the PubMed, Web of Science, and Latin American and Caribbean Health Sciences Literature (*Literatura Latino-Americana e do Caribe em Ciências da Saúde* - Lilacs) databases, using the keywords “inflammatory bowel disease,” “diet,” and “diet therapy” in English and Portuguese, with filters for pediatric studies published in the previous five years.

**Data summary:**

We selected 16 articles for this study, nine on exclusive and/or partial enteral nutrition and seven on modified oral diets, such as the specific carbohydrate diet (SCD) and the Crohn’s Disease exclusion diet (CDED). The studies found evaluated the anthropometric profile of patients and the inflammatory profile of diseases in children before and after the introduction of each specific nutrition therapy. All interventions presented positive changes in these parameters; however, the results were inconclusive regarding the efficacy of SCD and CDED in the treatment and prevention of relapses.

**Conclusions:**

Exclusive enteral nutrition has proven to be effective in inducing remission of Crohn’s Disease, and the use of partial enteral nutrition for maintenance treatment has shown promising results. Other modified oral diets are inconclusive concerning their effectiveness, requiring further randomized controlled clinical trials.

## RESUMO

**Objetivos:**

Revisar a literatura quanto à terapia nutricional oral e enteral e verificar evidências de sua eficácia tanto para tratamento quanto para prevenção de recidivas e redução de sintomas das doenças inflamatórias intestinais em pediatria.

**Fonte de dados:**

Foi realizada pesquisa bibliográfica nas bases de dados PubMed, Web of Science e Literatura Latino-Americana e do Caribe em Ciências da Saúde (Lilacs) utilizando os seguintes descritores em inglês: *inflammatory bowel disease*, *diet* e *diet therapy*; e os seguintes descritores em português: *doenças inflamatórias intestinais* e *dietoterapia*, com os filtros de estudo em Pediatria e no máximo cinco anos de publicação.

**Síntese dos dados:**

Foram selecionados 16 artigos para este estudo, sendo nove sobre o uso de nutrição enteral exclusiva e/ou parcial e sete sobre modificações da dieta oral, como a dieta específica de carboidratos (SCD) e a dieta de exclusão na Doença de Crohn (CDED). Os estudos encontrados avaliaram o perfil antropométrico dos pacientes e o perfil inflamatório das doenças em crianças antes e depois da introdução de cada terapia nutricional específica. Em todas as intervenções, foram observadas mudanças positivas nesses parâmetros, entretanto os resultados mostraram-se inconclusivos em relação à eficácia da SCD e da CDED no tratamento e na prevenção de recidivas.

**Conclusões:**

A nutrição enteral exclusiva mostrou-se uma terapia eficaz para a indução da remissão na Doença de Crohn, e o uso da nutrição enteral parcial para tratamento de manutenção vem exibindo resultados promissores. As demais modificações de dietas orais são inconclusivas a respeito de sua eficácia, sendo necessários mais ensaios clínicos randomizados e controlados.

## INTRODUCTION

The chronicity of the inflammation of the gastrointestinal tract (GIT) interferes directly with the quality of life and the nutritional status of individuals with inflammatory bowel diseases (IBD). Eating habits are believed to be involved in inducing inflammation, as well as in its remission.^1^ The hypothesis suggests that food - or the exclusion of some items - can modulate the GIT response to the inflammation, favoring a healthy intestinal microbiota.^2^ Therefore, dietary interventions would be good treatment options, both from the medical and the patient’s point of view.

Dietary therapies that involve enteral nutrition (EN), modification of carbohydrates, and dietary fibers have been discussed and prescribed in the pediatric field as therapeutic and preventive proposals for Crohn’s Disease (CD) and ulcerative colitis (UC) inflammatory episodes.^3^Thus, a critical bibliographical survey on the existing dietary treatments and their outcomes is necessary to guide the health professional in their therapeutic choice.

This work aimed to review the literature on oral and enteral nutrition therapy and investigate the evidence of its efficacy as a treatment, as well as in preventing relapses and reducing symptoms of IBD in the pediatric population.

## METHOD

Between May and August 2018, we performed a bibliographic search in the PubMed, Web of Science, and Latin American and Caribbean Health Sciences Literature (*Literatura Latino-Americana e do Caribe em Ciências da Saúde* - Lilacs) databases, using the keywords “inflammatory bowel disease,” “diet,” and “diet therapy” in English and Portuguese, with filters for pediatric studies published in the previous five years. We included articles in English, Spanish, and Portuguese.

After sorting the articles by reading their titles and abstracts, we excluded those that did not use a nutritional intervention, case studies, letters to the editor, reviews, studies conducted exclusively with adults, duplicate works, and mixed studies of children and adults with more than one-third of the population consisting of adults or with an age difference higher than 20 years.

### Dietary interventions in inflammatory bowel diseases

**Figure 1:**
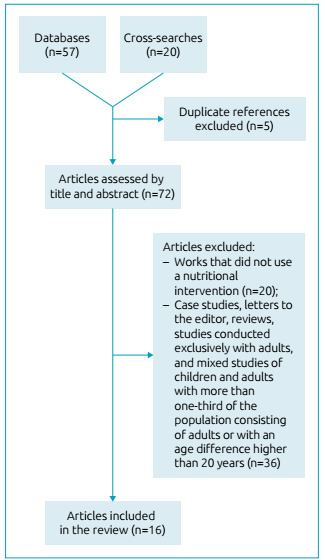
Article selection flowchart.

We found 57 articles in the databases and 20 in cross-searches. After the exclusions, 16 articles remained in this study, all in English (Figure 1). Among these 16 articles, nine addressed the use of exclusive and/or partial enteral nutrition, and seven covered modified oral diets.

### Exclusive enteral nutrition

Exclusive enteral nutrition (EEN) has been widely studied and recommended as first-line therapy for inducing remission of luminal CD. It consists of offering a nutritionally complete liquid diet, either polymeric or oligomeric (semi-elemental or elemental), exclusively, for six to eight weeks, through a nasogastric tube or orally.^4^ ^,^ ^5^

Most of the studies evaluated recommended the use of polymeric formulas, saving the elemental ones for those who do not tolerate diets with intact protein. Oral administration was preferable, but patients who did not like the taste of the formula or who were unable to feed orally received it through a tube.^6^ ^,^ ^7^ ^,^ ^8^ ^,^ ^9^ ^,^ ^10^ ^,^ ^11^ ^,^ ^12^

When compared to systemic corticosteroids or anti-TNF (immunomodulatory agent), EEN seems to be as effective as these drugs in inducing clinical remission of CD, characterized by the reduction in the disease activity index (Pediatric Crohn’s Disease Activity Index *-* PCDAI).^6^ ^,^ ^8^ ^,^ ^10^ ^,^ ^12^ In addition, there are advantages in minimizing adverse effects, improving the nutritional status, and promoting the recovery of the intestinal mucosa in children, analyzed by fecal calprotectin levels, which stands out as a sensitive inflammatory marker of the intestinal mucosa and is well correlated with endoscopic findings.^6^ ^,^ ^7^ ^,^ ^10^ ^,^ ^11^ ^,^ ^12^ ^,^ ^13^ ^,^ ^14^ Although the clinical improvement is similar to that of the different therapies, the studies presented no comparison of endoscopic mucosal remission.

In 2013, Soo et al. compared the bone mineral density (BMD) adjusted for age and height of a group that received EEN with another that received corticosteroids as therapy for inducing remission for six to eight weeks, using dual-energy X-ray absorptiometry (DEXA). They analyzed the changes in BMD (at the beginning of the study and after 12 to 18 weeks of follow-up) in both groups, and, despite the lack of statistical difference, the z-score variation was greater in patients who received EEN compared to those who received corticosteroids. The authors suggested that the result probably has clinical relevance since the change in BMD was close to 0 in the group that received corticosteroids and declared that long-term use of high doses of this medicine could cause growth damage and bone mass loss, especially in adolescents. In the same study, the remission and relapse rates showed no difference between the groups after a year of follow-up, but the group that received EEN had an improvement in weight-for-age.^12^

In 2014, after patients with CD achieved remission with the use of EEN or corticosteroids for six to eight weeks, Hojsak et al. analyzed the influence of a few factors (such as age, weight-for-height, and use of medicines and EEN) during the remission period and found that, after a year, only EEN was a protective factor against relapse. Moreover, the duration of remission was significantly higher in patients who received EEN as an induction therapy compared to those who used corticosteroids.^8^

Regarding the duration of EEN, the European Society for Paediatric Gastroenterology, Hepatology and Nutrition (ESPGHAN) recommends at least six weeks of use, even though the mucosa usually heals after eight weeks.^5^ All studies analyzed in this review indicated six to eight weeks of EEN as protocol, and most of them encouraged the participants to complete the eight weeks. In 2013, De Bie et al. used the nutrition therapy for only six weeks and revealed the effectiveness of the diet with respect to remission, but they identified an increase in symptoms in the first few weeks after the end of the EEN treatment and high rates of relapse despite the frequent use of azathioprine (immunosuppressive agent) as maintenance therapy, suggesting that long-term use of EEN could be more beneficial.^14^

Clinical and laboratory aspects showed that EEN significantly improved weight, albumin, erythrocyte sedimentation rate (ESR), hemoglobin, and hematocrit, with a decrease in inflammatory markers such as C-reactive protein (CRP) and fecal calprotectin levels.^7^ ^,^ ^10^ ^,^ ^14^

Grover et al. found that EEN was also able to promote good endoscopic response with reduced rates of relapse, hospitalization, need for anti-TNF, and surgical resection one year after remission.^11^

Despite the complexity of the pathogenesis of IBD, some studies show a direct relationship with dysbiosis, mainly because of the deregulated induction of the immune system, which reinforces the need for dietary interventions that act as protective factors against intestinal inflammation.^15^ ^,^ ^16^ ^,^ ^17^ The mechanisms by which the EEN acts in the intestine, however, are not yet well understood. Some studies indicate a change in the intestinal microbiota, reduction in mucosal exposure to food antigens from a conventional diet, decreased intestinal synthesis of inflammatory mediators due to lower fat supply, and increased supply of micronutrients to the inflamed bowel.^7^ ^,^ ^9^ ^,^ ^13^

Another unclear aspect is whether the location of the disease influences the outcome of nutritional treatment. Currently, EEN is recommended for any topography of luminal CD.^5^

According to ESPGHAN, EEN is not indicated for patients with UC.^5^ The European Society for Clinical Nutrition and Metabolism (ESPEN) declares that this diet seems safe, recommending it only as an adjuvant therapy to the standard nutritional treatment in patients with severe UC.^4^ We found no recent studies on the use of EEN in pediatric patients with UC.

### Partial enteral nutrition

Partial enteral nutrition (PEN) consists of providing a nutritionally balanced liquid formula to supplement a diet composed of solid foods (unrestricted or exclusion). PEN is being studied as maintenance therapy to prolong the remission of CD, but there is still no consensus or recommendation about its use.^4^ ^,^ ^5^

In 2015, Konno et al. revealed that the intake of at least 30 kcal/kg/day of elemental diet as maintenance therapy (after remission induction) showed a protective effect against relapse, controlling complications and delaying the need for surgery and the use of drugs (corticosteroids, immunosuppressive agents, and anti-TNF), with no difference regarding location or phenotype of the disease. Nevertheless, the diet was combined with aminosalicylates, which modulate the secretion of pro-inflammatory cytokines, leading to a bias for the outcome.^13^

In 2014, Duncan et al. used EEN to induce remission of CD, and after eight weeks, they encouraged the patients to consume 25% of the initial volume of the diet (polymeric or oligomeric, depending on the patient’s condition) until the end of one year of follow-up. After six months, clinical remission was more significant in patients who received PEN alone as maintenance treatment compared to the group that received no treatment (6/6 vs. 2/13, respectively, p=0.003). The group that received PEN+azathioprine presented a remission rate three times higher than the group without treatment. At the end of a year of follow-up, the use of PEN was comparable to that of azathioprine. Despite the promising results, the low adherence to PEN (31% of participants) shows the limiting factor of palatability and the monotony of taste in this modality of treatment.^9^

In 2018, Gavin et al. demonstrated that the use of PEN as maintenance treatment for four months after remission led to a relapse rate similar to that in patients on a conventional diet. Besides, EN increased the risk of overweight in patients.^6^

A prospective cohort study conducted in Canada and the United States divided 90 patients into three groups according to remission induction therapy: PEN, EEN, and anti-TNF. At the end of eight weeks, the results were positive for EEN and anti-TNF regarding the mucosal healing, and EEN has proven superior to PEN as to the quality of life and reduction in intestinal inflammation, evaluated by the decrease in fecal calprotectin levels, even though the energy intake was higher in the group that received PEN.^10^

Despite the promising results concerning the relapse time, further studies are necessary to ratify the indication of PEN and establish the optimal dosage and duration for maintenance of remission. For now, this type of treatment is an option to help maintain the remission in patients with mild diseases and low risk of relapse, but the evidence is limited as to its use in Pediatrics, and it is not indicated as a monotherapy in the maintenance of remission of CD.^5^

Most of the studies found about EN presented limitations, such as retrospective design, single-center research, and possible data loss, as the information was gathered from medical records. In addition, the sample population was heterogeneous, and a large part of the participants received concomitant drug treatment, which suggests the need for prospective multicenter studies, such as controlled clinical trials, with stratified randomization (according to the use of medication).

### Specific carbohydrate diet

The specific carbohydrate diet (SCD) was first described in 1920 by Hass as a therapy for celiac disease and was later studied for IBD.^18^ It consists of restricting most carbohydrates (such as starch, poly- and disaccharides - except monosaccharides) and increasing the consumption of proteins and fats, as poly- and disaccharides are believed to be involved in inducing an inflammatory response and acidity in the GIT, given that the poor absorption of these carbohydrates results in significant growth of bacteria and yeasts.^19^

Some studies showed a positive association between SCD and IBD, with reduced symptoms and inflammation and changes in anthropometric patterns. We found five pediatric studies that described the use of SCD as dietary treatment and its possible outcomes.

In 2016, Obih et al. reviewed the medical records of 26 children with IBD who received SCD concomitantly with drug treatment with immunosuppressive agents; among these records, only six had UC as the diagnosis. The children were monitored for 24 months on their biochemical parameters of ESR, CRP, albumin (as inflammatory markers), and hematocrit (as a marker for anemia), in addition to the anthropometric assessment, including body mass index (BMI) and growth velocity (GV). Patients diagnosed with CD or UC presented an improvement in their serum inflammatory markers, with a reduction in ESR and CRP and an increase in albumin. Their anemia (with increased hematocrit) and nutritional status (higher BMI and normal GV) also improved. However, out of the six patients with UC, only three responded positively to SCD (the other three were discontinued from the study because they did not show a satisfactory outcome, returning to the conventional treatment). Although SCD has proven to be positive for both pathologies, the sample size of UC was relatively small when compared with CD.^20^

Another study analyzed the exclusive use of SCD in seven patients with CD, adopting the same biochemical and anthropometric parameters. The authors also identified an improvement in the inflammatory response, with decreased CRP and increased albumin and hematocrit. Three months after the beginning of the diet, they detected weight gain and a reduction in clinical symptoms. The GV of the patients remained normal.^21^

Besides the biochemical, anthropometric, and clinical parameters, a study conducted in Atlanta with nine patients with CD evaluated the mucosal integrity (through endoscopy) and the inflammation (with the Lewis score) in patients who received SCD+drug treatment for 12 to 52 weeks. The anthropometric and biochemical parameters were similar to those of other studies. The researchers identified an improvement in the intestinal mucosa, with ulcer healing after 12 weeks of treatment. After the 52^nd^ week, only seven patients remained in the study, and they presented a reduction in the CD activity index; however, four patients had a higher Lewis score for mucosal inflammation than in the 12^th^ week. Among the other patients, two presented mucosal healing, and one continued to improve.^22^

Since the SCD is a restrictive diet that requires changes in the dietary pattern, making long-term adherence challenging to maintain, some protocols adopt the liberalization of the diet for a certain period (when the symptoms and biochemical parameters are stable) at the request of the patient and based on their food preferences. This liberalization usually consists of a modified version of the SCD (mSCD), with the introduction of one or two restricted foods per week or day. Nevertheless, no consensus was reached about the prescription of this protocol due to the lack of evidence of its effectiveness in the remission of inflammation and symptoms of the disease.

In 2016, Burgis et al. analyzed the effects of SCD for 12 months and the impacts after eight months of mSCD in 11 patients. The study showed an improvement in the levels of albumin, ESR, and hematocrit, as well as in anthropometric parameters, even after the mSCD. The patients gained weight, with a small loss without statistical significance after the liberalization.^23^

In 2017, Wahbeh et al. assessed biochemical and anthropometric parameters, as well as mucosal healing in the upper and lower GIT (with endoscopy) in seven patients who received both SCD and mSCD. They found that, in the two diets, most patients maintained the CRP within the normal range, with slightly abnormal levels of albumin, hematocrit, and fecal calprotectin. BMI did not change, and the authors did not detect mucosal healing in the patients.^24^

Despite the positive results of the presented studies, the authors highlight that they are not conclusive regarding whether these diets ensure the remission of IBD. Factors such as the small sample size, the retrospective design of most studies, and the combination of diet and drug treatment prevent their recommendation. These limitations led ESPGHAN not to indicate this type of intervention for children with these diseases.^5^ Nonetheless, even though these dietary interventions are restrictive and inconclusive about their effects both on the induction and remission periods, the diet feasibility should be taken into account, not only from a medical but also from the patient’s point of view, as these interventions prioritize the oral diet, promoting all of its social benefits.

### Crohn’s Disease exclusion diet

Crohn’s Disease exclusion diet (CDED) consists of offering fruits and vegetables, some types of meat, and carbohydrates and restricting or excluding the consumption of animal fat, processed meat (including fish), gluten, dairy, emulsifiers, canned foods, and some monosaccharides. Its use is recommended in combination with EN, which consists of a polymeric formula that provides 50% of the daily energy intake.^5^ ^,^ ^25^ ^,^ ^26^

The mechanisms by which this therapy works are not known, but one assumption is that the exclusion of some foods might decrease the bacterial translocation and prevent the pro-inflammatory action of some dietary components, facilitating the effect of medicines.^25^ ^,^ ^26^ There is no evidence for its use as a remission induction therapy.^5^

We found two recent pediatric studies on the use of CDED and EN performed by the same authors in Israel. In 2014, Sigall-Boneh et al. analyzed the use of CDED+EN for six weeks and obtained positive responses, such as most pediatric patients reaching clinical remission and improvement in the PCDAI, CRP, ESR, albumin, and weight after 12 weeks of follow-up in individuals with mild or moderate diseases. The institutional standard demanded that all patients used immunomodulatory agents; therefore, whether the diets led to remission induction remains unclear.^25^

In 2017, Sigall-Boneh et al. evaluated the same nutrition therapy for the same period and found that this strategy can induce remission of CD or, at least, favor clinical response, as evidenced by the drop in the Harvey-Bradshaw index (HBI) and inflammatory markers. According to the authors, the positive outcomes resulted from a reduction in intestinal exposure to inflammatory triggering factors. However, as in the other study, these findings have a bias, as all patients used anti-TNF since the start of treatment.^26^

The small number of longitudinal studies on the use of CDED is a limiting factor for any kind of indication. Nevertheless, some studies have shown associations between low risk of developing IBD and a diet with a high intake of fiber, fruits, and vegetables and between high risk of IBD and a diet rich in linoleic acid, animal fat and protein, and refined sugar. Since the Western diets have risk characteristics for the development of IBD, we can assume that diets which exclude or restrict certain types of food, such as CDED or semi-vegetarian diets, can help reduce symptoms and prolong remission, although more studies are necessary to validate their use.^3^ ^,^ ^27^ ^,^ ^28^ ^,^ ^29^

### Low fermentable oligo-, di-, monosaccharides and polyol diet

The discussion about fermentable oligo-, di-, monosaccharides, and polyols (FODMAP) in IBD (low content of fermentable carbohydrates, such as oligosaccharides, disaccharides, monosaccharides, and polyols)^30^ is based on two hypotheses that involve their digestion. FODMAPs are believed to be osmotically active molecules, leading to an increase in intraluminal water in the small intestine, abdominal distension, and, consequently, higher orocecal transit, hindering their absorption. Another hypothesis is that the FODMAPs reach the part of the colon that does not absorb them, resulting in rapid fermentation by colonic bacteria, which causes flatulence, swelling, and discomfort due to the increased production of gas and distension.^31^ ^,^ ^32^ Thus, a diet with low FODMAP content would be beneficial in controlling the symptoms. Despite these speculations, we found no recent study about this diet in children with IBD, and ESPGHAN does not recommend its use.^5^

### Supplementation in inflammatory bowel diseases

Experts claim that no specific diet exits for the remission stage of IBD; however, some strategies can help, such as the use of the strain of *Escherichia coli* Nissle, 1917, and the VSL#3 (mixture of lactic acid bacteria and bifidobacteria). These probiotics can be considered in the maintenance of remission in patients with UC.^4^ ^,^ ^5^

In addition, ESPEN states that probiotics are effective in inducing remission of mild to moderate UC. ESPGHAN indicates that the evidence is limited but favorable to the use of probiotics combined with the standard treatment for inducing remission in pediatric patients with UC. None of these societies indicates their use in CD.^4^ ^,^ ^5^

There is no recommendation for the use of prebiotics, synbiotics, or omega-3 in IBD. On the other hand, the intake of dietary fiber is good for improving gastrointestinal functions and might be effective in UC; when associated with the standard therapy, it can help the maintenance treatment of the disease. Fiber restriction should not be recommended for patients with IBD, except for those who have the stenotic phenotype of the disease.^4^ ^,^ ^5^

The literature has described the supplementation of curcumin as a strategy for the treatment of IBD. Its anti-inflammatory and antioxidant action might lead to a remission of UC,^33^ ^,^ ^34^ ^,^ ^35^ as well as a reduction in symptoms and inflammatory markers of CD.^36^ Nonetheless, no consensus has been reached about its nutraceutical potential, or the safe dosage recommended.^37^ ESPGHAN declares that curcumin may be considered in the treatment of UC, both for induction and remission, but establishing a safe supplementation dose in Pediatrics is still necessary.^38^

We found no recent studies about supplementation in IBD.

The study of oral and enteral nutrition therapy in IBD is complex and has been increasingly discussed. EEN continues to be as effective as drugs for remission induction in CD among pediatric patients, and the use of PEN as maintenance treatment has become more relevant, particularly due to the possibility of decreasing the use of medicines at this stage of the disease.

In this review, we found few original and recent articles, which presented small samples and some methodological biases, as discussed throughout the work. These limitations did not allow us to recommend oral diets for inducing remission or preventing relapses in patients with IBD, mainly because of the concomitant use of medication during the treatment. These interventions might assist in the control of IBD symptoms, but randomized controlled clinical trials are necessary to establish safe and effective recommendations for the pediatric population.
